# Adaptive immune responses in vaccinated patients with symptomatic SARS-CoV-2 Alpha infection

**DOI:** 10.1172/jci.insight.155944

**Published:** 2022-03-08

**Authors:** Han-Sol Park, Janna R. Shapiro, Ioannis Sitaras, Bezawit A. Woldemeskel, Caroline C. Garliss, Amanda Dziedzic, Jaiprasath Sachithanandham, Anne E. Jedlicka, Christopher A. Caputo, Kimberly E. Rousseau, Manjusha Thakar, San Suwanmanee, Pricila Hauk, Lateef Aliyu, Natalia I. Majewska, Sushmita Koley, Bela Patel, Patrick Broderick, Giselle Mosnaim, Sonya L. Heath, Emily S. Spivak, Aarthi Shenoy, Evan M. Bloch, Thomas J. Gniadek, Shmuel Shoham, Arturo Casadevall, Daniel Hanley, Andrea L. Cox, Oliver Laeyendecker, Michael J. Betenbaugh, Steven M. Cramer, Heba H. Mostafa, Andrew Pekosz, Joel N. Blankson, Sabra L. Klein, Aaron A.R. Tobian, David Sullivan, Kelly A. Gebo

**Affiliations:** 1 W. Harry Feinstone Department of Molecular Microbiology and Immunology and; 2 Department of International Health, Johns Hopkins Bloomberg School of Public Health, Baltimore, Maryland, USA.; 3 Department of Medicine, Division of Infectious Diseases, Johns Hopkins School of Medicine, Baltimore, Maryland, USA.; 4 Advanced Mammalian Biomanufacturing Innovation Center, Department of Chemical and Biomolecular Engineering, Johns Hopkins University, Baltimore, Maryland, USA.; 5 Department of Chemical and Biological Engineering and Center for Biotechnology and Interdisciplinary Studies, Rensselaer Polytechnic Institute, Troy, New York, USA.; 6 Critical Care Medicine, University of Texas Health, Houston, Texas, USA.; 7 Emergency Medicine, Danbury Connecticut, USA.; 8 Division of Allergy and Immunology, Department of Medicine, Northshore University Health System, Evanston, Illinois, USA.; 9 Department of Medicine, Division of Infectious Diseases, University of Alabama Birmingham, Alabama, USA.; 10 Department of Medicine, Division of Infectious Diseases, University of Utah, Salt Lake City, Utah, USA.; 11 Hematology Oncology, Medstar Washington Hospital Center, Washington, DC, USA.; 12 Department of Pathology, Johns Hopkins School of Medicine, Baltimore, Maryland, USA.; 13 Department of Pathology and Laboratory Medicine, Northshore University Health System, Evanston, Illinois, USA.; 14 Department of Neurology, Johns Hopkins School of Medicine, Baltimore, Maryland, USA.; 15 Division of Intramural Research, National Institute of Allergy and Infectious Diseases, NIH, Bethesda, Maryland, USA.; 16 Department of Epidemiology, Johns Hopkins Bloomberg School of Public Health, Baltimore, Maryland, USA.

**Keywords:** COVID-19, Infectious disease, Adaptive immunity, Cellular immune response, Genetic variation

## Abstract

Benchmarks for protective immunity from infection or severe disease after SARS-CoV-2 vaccination are still being defined. Here, we characterized virus neutralizing and ELISA antibody levels, cellular immune responses, and viral variants in 4 separate groups: healthy controls (HCs) weeks (early) or months (late) following vaccination in comparison with symptomatic patients with SARS-CoV-2 after partial or full mRNA vaccination. During the period of the study, most symptomatic breakthrough infections were caused by the SARS-CoV-2 Alpha variant. Neutralizing antibody levels in the HCs were sustained over time against the vaccine parent virus but decreased against the Alpha variant, whereas IgG titers and T cell responses against the parent virus and Alpha variant declined over time. Both partially and fully vaccinated patients with symptomatic infections had lower virus neutralizing antibody levels against the parent virus than the HCs, similar IgG antibody titers, and similar virus-specific T cell responses measured by IFN-γ. Compared with HCs, neutralization activity against the Alpha variant was lower in the partially vaccinated infected patients and tended to be lower in the fully vaccinated infected patients. In this cohort of breakthrough infections, parent virus neutralization was the superior predictor of breakthrough infections with the Alpha variant of SARS-CoV-2.

## Introduction

The mRNA COVID-19 vaccines are 90% effective at preventing severe disease leading to hospitalization, which includes infections with the Alpha and Delta variants (1–3) of the SARS-CoV-2 virus. The United States had 10,262 documented vaccine breakthrough cases from January 1 to April 30, 2021, out of approximately 101 million fully vaccinated individuals (4). During this same period, 706 vaccine breakthrough cases resulted in hospitalization for COVID-19, and of these, 132 were fatal. As of May 1, 2021, the CDC switched to counting only hospitalizations and deaths resulting from vaccine breakthrough cases (5). The total number of people hospitalized in the US after receiving a BNT162b2, mRNA-1273, or Ad26.COV2.S SARS-CoV-2 vaccine by August 31, 2021, was 10,741 (48% female), with 7,282 over age 65 (5). An analysis of vaccine breakthroughs from the United Kingdom from December 2020 to July 2021, when the Alpha variant predominated, showed that breakthrough infections in vaccinated people result in fewer COVID-19 symptoms, shorter duration of symptoms, less frequent hospitalizations, and more asymptomatic infections than in unvaccinated people (6).

Benchmarks for humoral or cellular immunity that translate to protection against SARS-CoV-2 asymptomatic infection, symptomatic COVID-19, hospitalization, or death are still being defined. The circulation of SARS-CoV-2 variants further complicates the establishment of such benchmarks due to sequence differences compared with the vaccine-seed strains (i.e., parent virus). Many studies show that high antibody titers in the plasma of convalescent or fully vaccinated individuals can adequately neutralize most SARS-CoV-2 variants, suggesting that if individuals develop sufficiently high levels of antiviral antibody, the vaccine can protect against severe disease (7–9). Less data exist on correlates of protection conferred by cellular immune responses against SARS-CoV-2 in unvaccinated people (10) compared with vaccinated people (11) in the context of antibody responses (12).

During an outpatient trial to evaluate the efficacy of convalescent plasma administered early in infection, we identified vaccinated individuals with confirmed SARS-CoV-2 infection (i.e., breakthrough cases). We conducted a study to compare humoral and cellular responses in infected patients at least 2weeks following a first dose (partially vaccinated) or second dose (fully vaccinated) of a SARS-CoV-2 mRNA vaccine to the responses of fully vaccinated healthy controls (HCs). After sequencing the infecting viruses, we compared measures of humoral and cellular immunity to both a parent strain with a spike protein similar to the one used in the mRNA vaccines and the Alpha variant of SARS-CoV-2 in the infected individuals to healthy vaccinated controls. We report that vaccinated patients with confirmed symptomatic SARS-CoV-2 infections either after the first or second dose had similarly high IgG antibody titers and cell-mediated immune responses, but significantly lower virus neutralizing antibody (nAb) levels compared with healthy vaccinated controls, suggesting that a reduced nAb level is a key factor in greater susceptibility to breakthrough infection.

## Results

### Study population.

This study population, described in Table 1, included samples from 4 separate patient groups: a) vaccinated uninfected HCs sampled 7–14 days after vaccination (early fully vaccinated HC [Early FV-HC]); b) vaccinated uninfected HCs sampled approximately 95–187 days after vaccination (Late FV-HC); c) partially vaccinated (PV) patients with symptomatic confirmed SARS-CoV-2 infection (PV-I); and d) fully vaccinated (FV) patients with symptomatic confirmed SARS-CoV-2 infection (FV-I). All participants received mRNA-based vaccines, i.e., either BNT162b2 or mRNA-1273. The symptomatic breakthrough patients were identified and evaluated from January 4 to June 4, 2021, before the Delta variant became predominant.

Of the fully vaccinated HCs sampled early and late following vaccination, 49% were female. The majority (89%) of the HCs had received the BNT162b2 mRNA vaccine. Most of the PV-I patients (*n =* 22) were male (55%) with a median age of 46 years (IQR: 33–55) and had received the BNT162b2 vaccine (72.7%). The mean time from first vaccine dose to presentation with infection (i.e., sample collection) was 20 days (range 14–38). The main exposure was nonwork related, with onset of symptoms from 10 known point exposures averaging 2.4 days, and time from symptom onset to blood draw was 4 days (*n =* 13). Cough (81%), fatigue (77%), dyspnea on exertion/shortness of breath (55%), and altered taste or smell (55%) were the predominant symptoms, and a minority (42%) of patients had elevated C-reactive protein.

The majority of the 13 FV-I patients were female (69%), with a median age of 39 years (IQR 33–44). Over 3 quarters had received the BNT162b2 vaccine. The main exposure sources to SARS-CoV-2 were children or social activities such as travel or dining in a public venue. The median time from second vaccination to confirmed infection was 80 days (range 32–124). The mean time between point exposures and onset of symptoms from 6 known point exposures was 3.6 days and the mean time from symptom onset to screening visit blood draw was 4 days (*n =* 13). The most common symptoms were fatigue (77%), cough (77%), dyspnea on exertion (69%), and altered taste or smell (54%). The majority (85%) had elevated C-reactive protein. None of the infected patients were immunosuppressed or developed symptoms requiring hospitalization. Most reported being back to their normal healthy state within 2 weeks of symptom onset. Absolute lymphocytes were similar between PV-I (1.74k) and FV-I (1.79k) patients.

### SARS-CoV-2 Alpha variant caused a majority of infections in vaccinated individuals.

**Figure 1:**
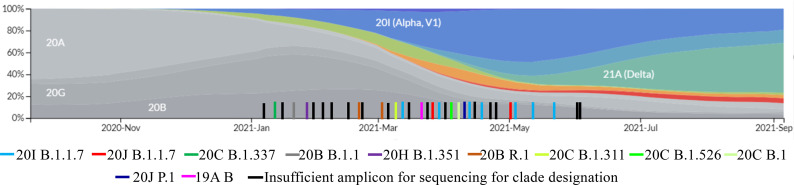
The incidence of majority breakthrough infections caused by SARS-CoV-2 variants and the frequency of SARS CoV2 circulating lineages in the USA between September 2020 and September 2021. Each bar with a specific color indicates the time when the breakthrough infections occurred. Data were retrieved from Nextstrain Genomic epidemiology of novel coronavirus North America-focused subsampling, further filtered data set by country-USA on September 27, 2021.

In the FV-I, the B.1.1.7 clade (Alpha variant) represented 7 out of 11 sequenced SARS-CoV-2 infections; P.1 (Gamma variant), B.1.526 (Iota variant), B.1.311, and early B.1 lineage (19A Nextstrain) accounted for the remaining 4. In the PV-I, only 8 samples yielded a successful sequence of the infecting viruses, which included 2 Alpha variants, 2 Gamma variants, and the remaining 4 consisted of various B.1 lineage viruses. The circulation of variants in the US over time is graphed in Figure 1.

### Humoral and cellular immune responses to SARS-CoV-2 variants in vaccinated HCs.

**Figure 2:**
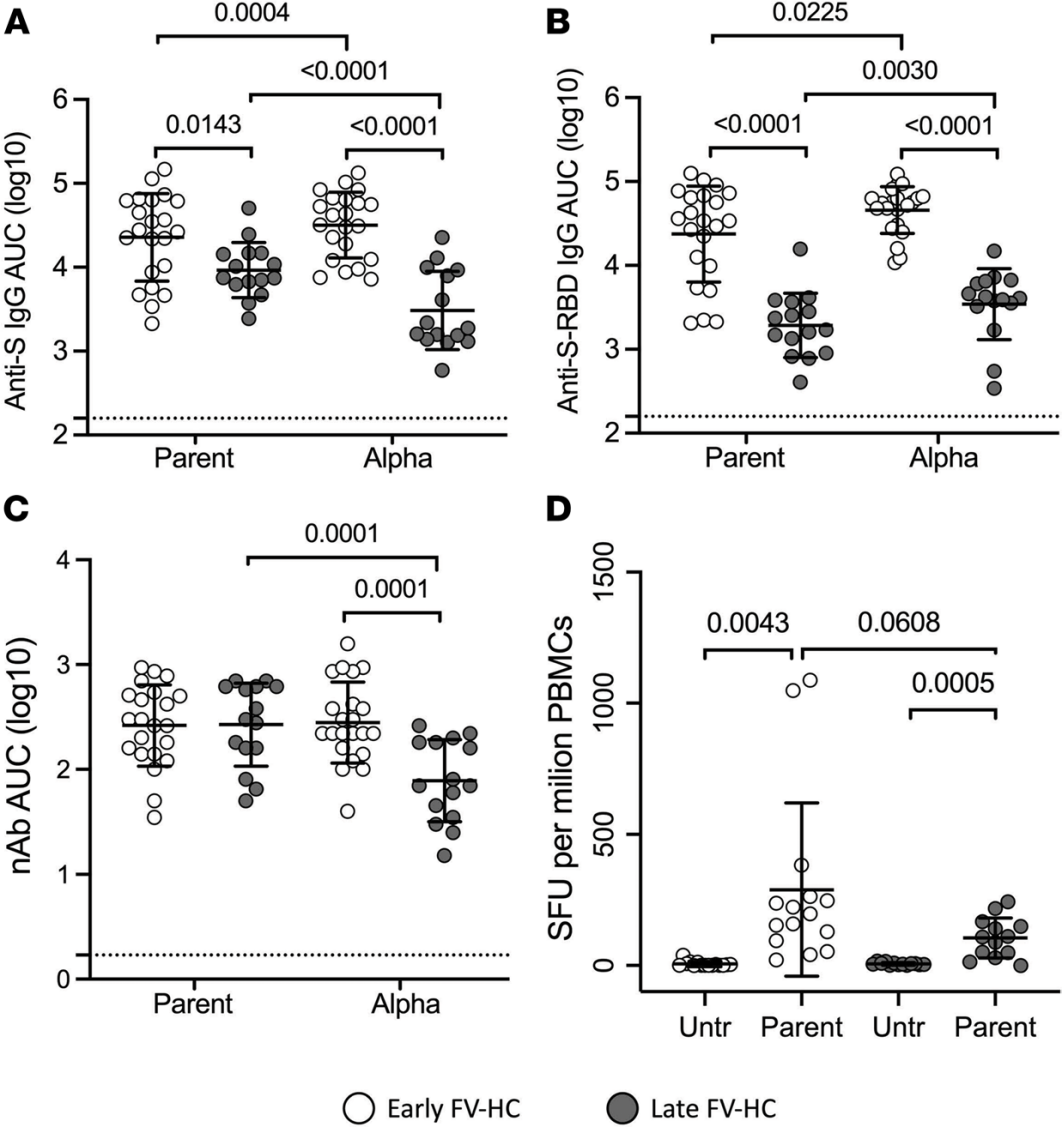
Measures of vaccine-induced humoral and cellular immunity decrease with time in HCs, with the exception of nAb to the parent strain. Plasma and PBMC samples were collected from fully vaccinated HCs, with no history of testing positive for COVID-19, either 7–14 days (early, *n =* 22) or 95–187 days (late, *n =* 15) after the second dose. Indirect ELISAs were used to measure IgG against S (**A**) and the S-RBD (**B**) from either the parent strain or Alpha variant viruses and are graphed as AUC values. (**C**) Microneutralization assays were also performed against the parent virus and Alpha variant, and AUC values are shown. (**D**) An IFN-γ ELISPOT was used to measure the SFUs per million PBMCs in response to SARS-CoV-2 S parent strain peptide pools. In **A**–**C**, the dashed lines indicate the limit of detection. Two-tailed, unpaired *t* tests were used to compare between groups, and paired 2-tailed *t* tests were used to compare outcomes on the same individuals. P values below 0.05 are shown, but since 4 comparisons were made in each panel, the Bonferroni correction for multiple comparisons suggests that only values below 0.0125 (i.e., 0.05/4) be considered statistically significant.

To assess the kinetics of the humoral immune response after vaccination, we compared plasma antibodies that bind (measured by indirect IgG ELISA) and neutralize (measured by microneutralization assay) SARS-CoV-2 in the early and late FV-HC groups. AUC values were calculated by plotting the OD values (ELISA) or protection from cytopathic effects (microneutralization) against serial dilutions. For the SARS-CoV-2 spike (S) and S-receptor binding domain (S-RBD), the specific IgG responses against both the parent strain and Alpha variant were significantly lower in the late FV-HC group compared with the early FV-HC group, suggesting that responses decrease with time following receipt of the second vaccine dose (Figure 2, A and B). The late FV-HC group showed a 9%, 23%, 25%, and 24% mean reduction from the initial values of IgG responses compared with the early FV-HC group for the parent strain S, Alpha variant S, parent strain S-RBD, and Alpha variant S-RBD, respectively (Figure 2, A and B). These reductions were all statistically significant, and the reduction in anti–S-RBD IgG was greater in magnitude than the reduction in anti-S IgG for both the parent and Alpha variant viruses. Furthermore, in early FV-HC, the IgG responses to the parent S and S-RBD were lower than the IgG responses to the Alpha variant S and S-RBD by 3% (*P =* 0.0004) and 7% (*P =* 0.0225), respectively. The late FV-HC group showed a 12% lower (*P <* 0.0001) IgG response to the Alpha variant S compared with the parent S, whereas the IgG response to the Alpha variant S-RBD was 8% higher (*P =* 0.0030) than the response to the parent S-RBD (Figure 2, A and B).

Microneutralization assays demonstrated a significant difference in nAb activity (as expressed by AUC) against the Alpha variant between the early FV-HC group and the late FV-HC group, with a 23% decrease in the late FV-HC group compared with early FV-HCs (Figure 2C). The difference in nAb AUC against the parent virus between early and late FV-HCs, however, was negligible, suggesting that the antibody response against the Alpha variant significantly waned over 4 months in contrast to minimal waning of the nAb response to the parent virus. This is consistent with the lower binding to the Alpha variant S protein in the FV-HC group (Figure 2, A and B), which may result from the loss of recognition of nAb epitopes in the N- and C-terminus of the Alpha variant Spike protein. This decreasing trend with time was confirmed when assessing antibody responses relative to the number of days after the second vaccination in the late FV-HC group (Supplemental Figure 1, C and F; supplemental material available online with this article; https://doi.org/10.1172/jci.insight.155944DS1). In parallel to the AUC values, geometric mean titers (GMT) are summarized for each group in Supplemental Table 2 and follow similar patterns as the AUC values.

Humoral and cellular immune responses are known to interact closely to provide protection against viral infection. We performed IFN-γ enzyme–linked immunosorbent spot (ELISpot) assays on PBMCs obtained from FV-HCs to quantify the frequency of virus-specific T cells (Figure 2D). Due to low sample availability, cellular assays were not completed for all participants. Exact sample sizes are detailed in Supplemental Table 1. As expected, treatment with the SARS-CoV-2 S peptide pool stimulated a significant T cell response at both early and late time points in HCs. The early FV-HC group elicited a stronger T cell response, with a median of 197 spot-forming units (SFU) per million cells against the SARS-CoV-2 parent strain S peptide pool as previously reported (11), compared with less than 110 SFUs per million cells in the late FV-HC group (Figure 2D). As with the antibody data, this decreasing trend with time was confirmed when assessing T cell responses relative to the number of days after the second vaccination in the late FV-HC group (Supplemental Figure 1G).

### Humoral and cellular immune responses to SARS-CoV-2 in infected but vaccinated individuals.

**Figure 3:**
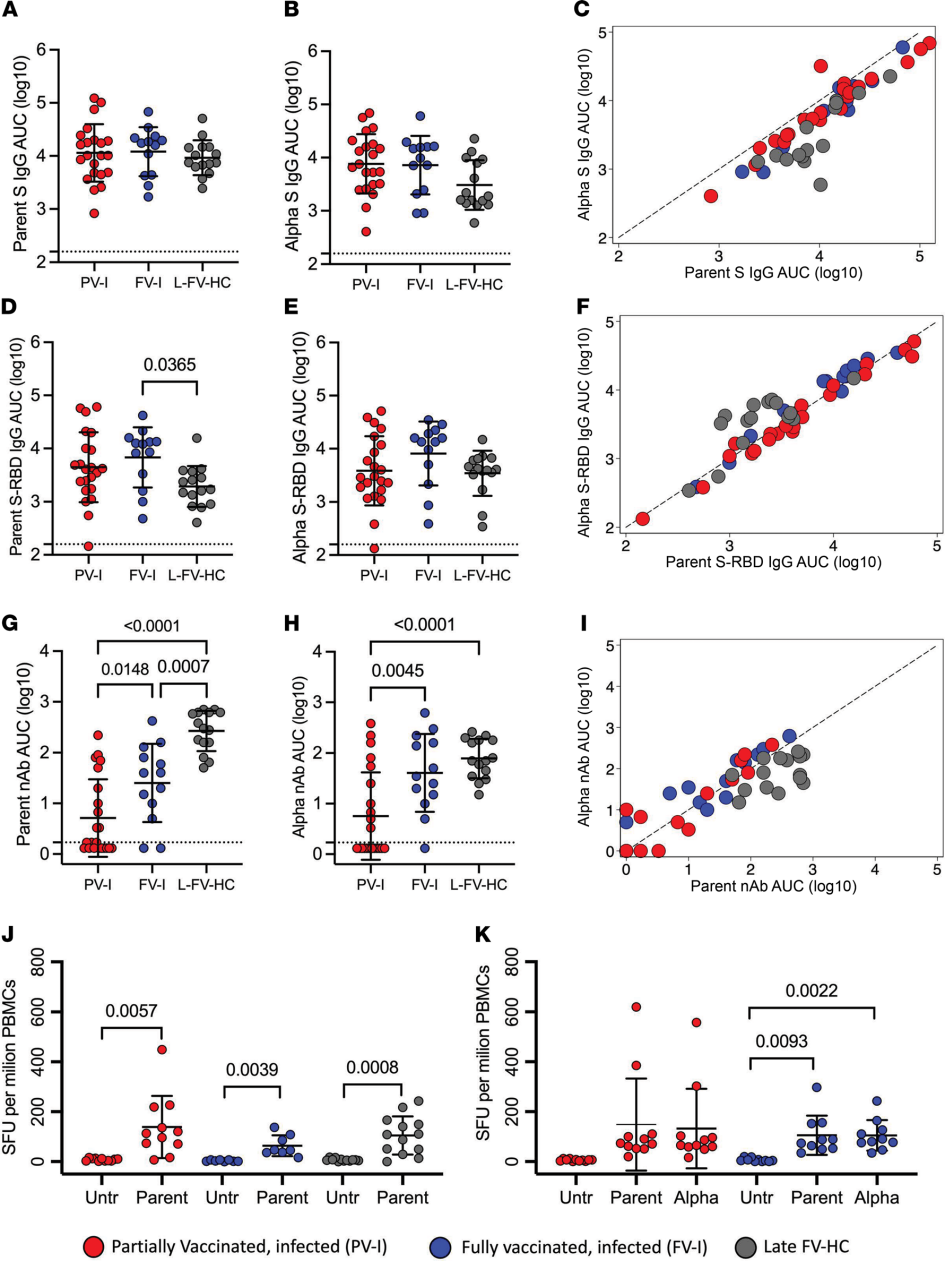
Antibody responses are greater in FV-I than FV-HCs. Plasma samples were collected from confirmed breakthrough infections that occurred either after receipt of the first vaccine dose (red circles, PV-I, *n =* 22) or after receipt of the second dose (blue circles, FV-I, *n =* 13). For comparison, plasma samples from fully vaccinated HCs were collected (grey circles, Late FV-HC, *n =* 15). Log_10_-transformed AUC values for anti-S IgG (indirect ELISA; **A**–**C**), anti-S-RBD IgG (indirect ELISA; **D**–**F**) and neutralizing antibodies (microneutralization assay; **G**–**I**) are shown for the 3 study groups for the parent virus (**A**, **D**, and **G**), the Alpha variant (**B**, **E**, and **H**), and as the correlation between the parent and Alpha variants (**C**, **F**, and **I**). Dashed lines indicate the limit of detection (**A**, **B**, **D**, **E**, **G**, and **H**) or the line of agreement (**C**, **F**, and **I**). (**J**) One-way ANOVA with Tukey’s correction for multiple comparisons were used to compare groups for antibody data, and paired 2-tailed *t* tests. (**K**) Repeated-measures ANOVA with Tukey’s correction for multiple comparisons were used to analyze ELISpot data. All *P* values below 0.05 are shown and were considered statically significant.

We compared antibody responses to SARS-CoV-2 in PV-I and FV-I patients with responses in the late FV-HC group using ELISA and microneutralization assays. The samples in the late FV-HC group were collected in a similar time frame relative to the second vaccine dose in the FV-I group (Supplemental Figure 1). Among the infected patients, there was no significant difference in IgG responses to SARS-CoV-2 S or S-RBD between PV-I and FV-I patients to either the parent or Alpha variant viruses (Figures 3, A, B, D, and E). The IgG responses to the parent strain and Alpha variant S and S-RBD tended to be higher in the infected groups than in the late FV-HC group and were significantly different for the fully vaccinated group in the case of the parent virus S-RBD (*P =* 0.0365; Figure 3D). The anti-S IgG AUC values were higher for the parent than Alpha variant virus (i.e., trending below the line of agreement) among the PV-I, FV-I, and HC groups (Figure 3C). In contrast, anti–S-RBD IgG AUC values were similar against the parent and Alpha variants (i.e., primarily on the line of agreement) (Figure 3F). Finally, anti-nucleocapsid IgG responses were not significantly different between HCs and either PV-I or FV-I patients (Supplemental Figure 2).

While IgG responses to SARS-CoV-2 antigens (parent and Alpha variant) were lower in the late FV-HC group compared with vaccinated and infected participants (Figure 3, A–F), the functional nAb response to the parent virus was significantly lower in the PV-I and FV-I group compared with that in the late FV-HC group (mean reduction of 73% and 43%, respectively; Figure 3G). Similarly, the nAb response to the Alpha variant was significantly lower in the PV-I group than in the late FV-HC group, but this difference was not significant for the FV-I group. nAb responses to the Alpha variant in the PV-I and FV-I groups were 63% (*P <* 0.0001) and 16% (ns) lower than those of the late FV-HC group, respectively (Figure 3H). Furthermore, the functional nAb responses against both the parent virus and the Alpha variant were significantly lower in the PV-I group than that in the FV-I group (52% and 58% lower in the PV-I group for Parent and Alpha, respectively) (Figures 3, G and H), supporting that full vaccination is important in eliciting nAb responses to SARS-CoV-2 variants of concern. Additional analysis showed a correlation between nAb responses against the parent virus and the Alpha variant (Figure 3I). Furthermore, patterns in AUC values were replicated on the GMT scale (Supplemental Table 2).

To determine if cell-mediated immunity was reduced in vaccinated patients with SARS-CoV-2 infections, T cell IFN-γ responses to the SARS-CoV-2 S peptide pool were evaluated. Like the HC group, cells from infected patients (i.e., PV-I and FV-I) treated with the peptide pool mounted a significantly greater IFN-γ response than untreated cells (Figure 3J). Further, the IFN-γ response was similar among the PV-I, FV-I, and the late FV-HC groups (Figure 3J). To determine if there was a reduction in cell-mediated immune responses to the Alpha variant in the vaccinated patients, we compared T cell responses to the parent and Alpha strains. There was no difference in T cell IFN-γ responses among either PV-I or FV-I to parent or Alpha S peptide pools (Figure 3K).

### Humoral and cell-mediated immune parameters are not associated in either HCs or vaccinated but infected patients.

**Figure 4:**
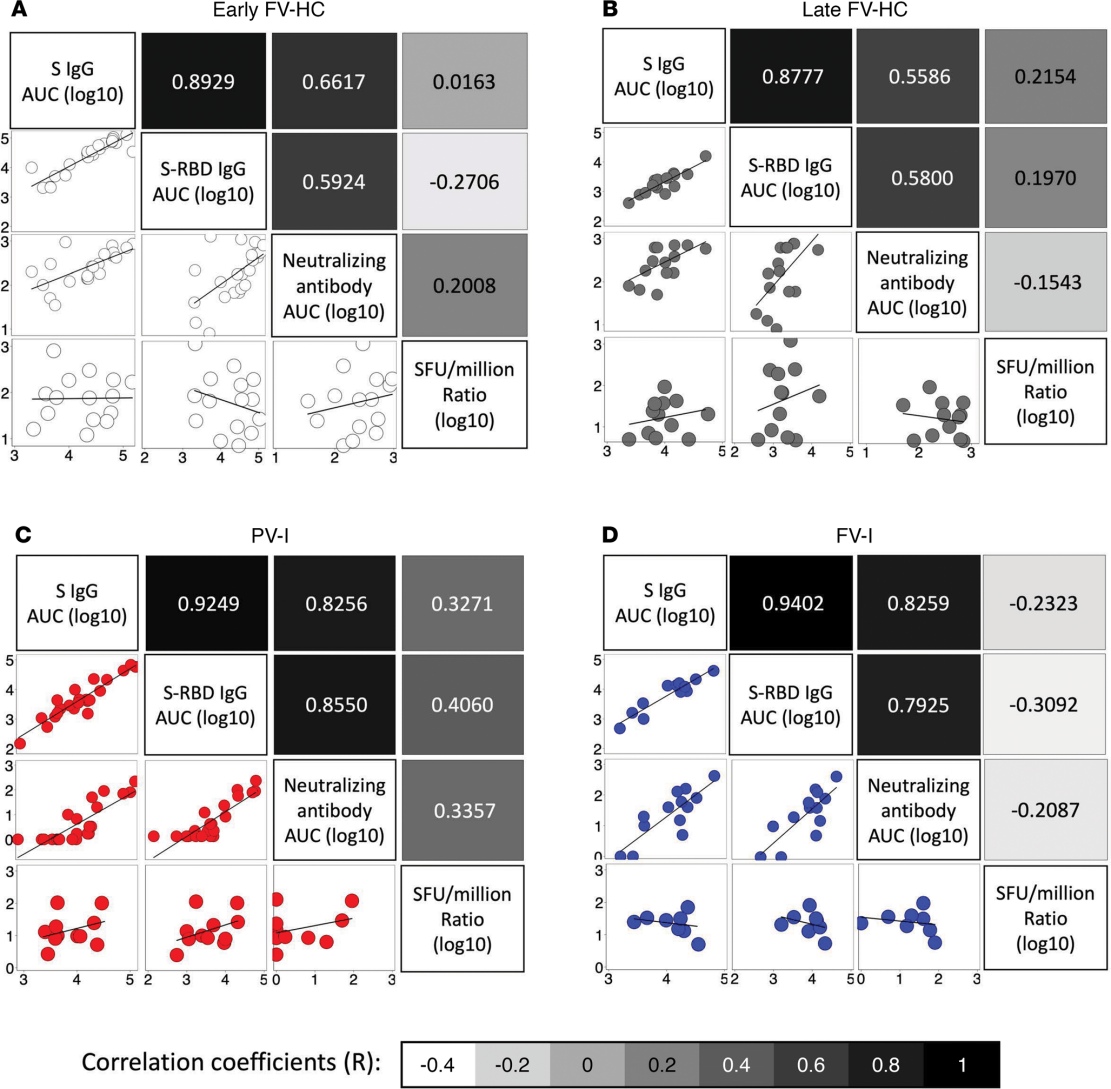
Antibody responses correlate well with each other but correlate poorly with measures of T cell-mediated immunity. The correlation between various measures of humoral and cell-mediated immunity were assessed separately as follows: (**A**) for HC sampled 7–14 days after vaccination (Early FV-HC); (**B**) for HC sampled 95–187 days after vaccination (Late FV-HC); (**C**) for individuals with confirmed SARS-CoV-2 after receipt of the first dose of a vaccine (PV-I); and (**D**) for individuals with confirmed SARS-CoV-2 after receipt of the second dose of a vaccine (FV-I). Scatter plots and trendlines are shown in the lower half of matrices, and correlation coefficients, color coded by the strength of the correlation, are shown in the upper half of the matrices. For cell-based measures, data shown is the ratio of SFUs per million for treated to untreated cells, transformed on a log_10_ scale.

**Figure 5:**
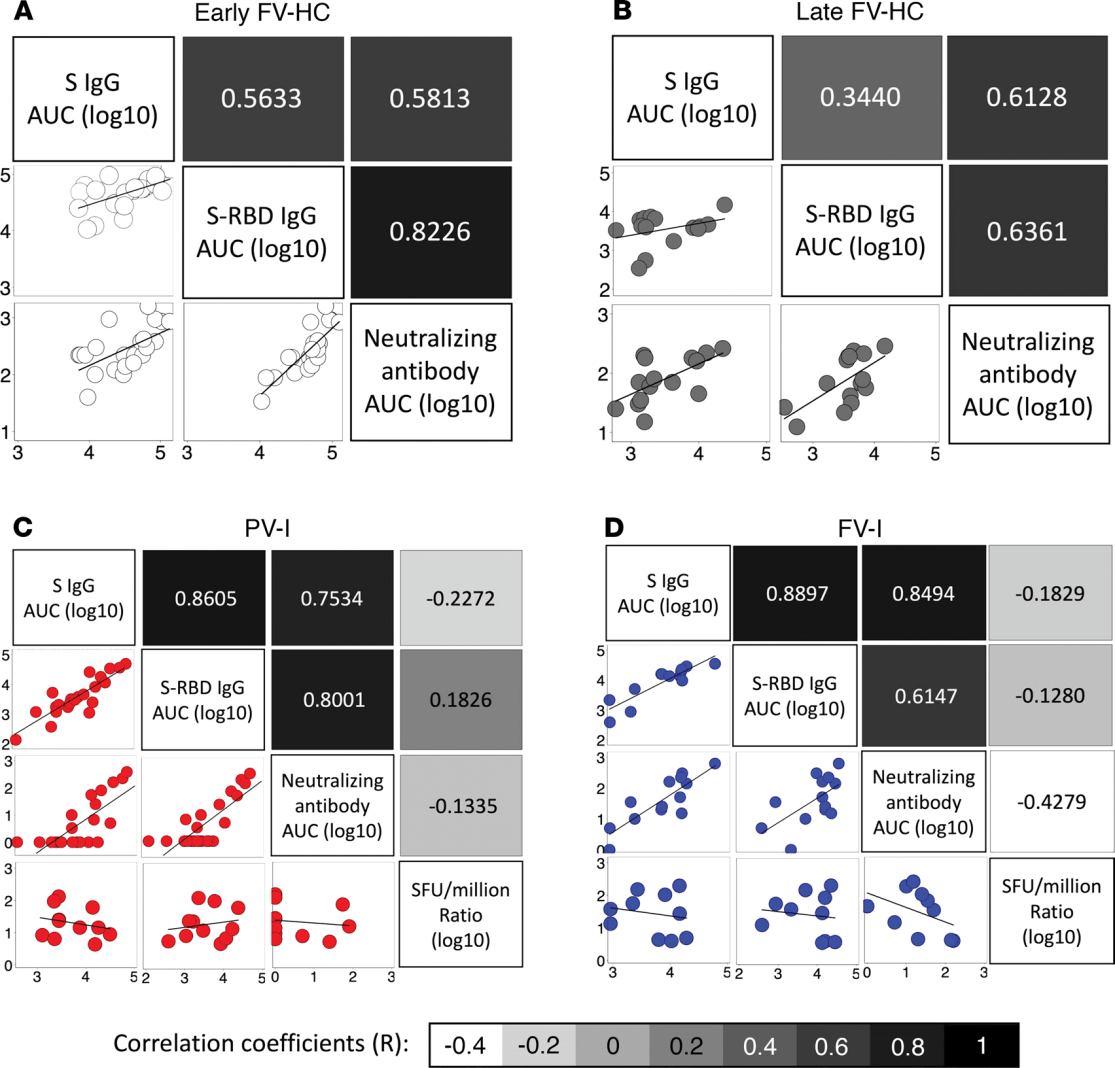
Antibody responses to the Alpha variant correlate well with each other but correlate poorly with measures of T cell-mediated immunity. The correlation between various measures of humoral and cell-mediated immunity to the Alpha variant were assessed separately for (**A**) early FV-HC; (**B**) late FV-HC; (**C**) PV-Is; and (**D**) FV-Is. Scatter plots and trendlines are shown in the lower half of matrices, and correlation coefficients, color coded by the strength of the correlation, are shown in the upper half of the matrices. For cell-based measures, data shown is the ratio of SFUs per million for treated to untreated cells, transformed on a log_10_ scale.

Within the HCs, PV-I, and FV-I patients, levels of binding IgG against either S or S-RBD and nAb to the parent strain all strongly correlated with each other, with the correlation coefficients (R) ranging from 0.55–0.94, indicating high levels of agreement between the 3 measures of humoral immunity (Figure 4, A–D). However, antibody responses were poorly correlated with measures of T cell-mediated immunity to the parent strain (R values ranging from –0.31 to 0.4; Figure 4, A–D). When assessing the correlation between cellular and humoral responses to the Alpha variant, similar trends were observed for the parent strain (Figure 5).

## Discussion

Symptomatic SARS-CoV-2 infections in vaccinated individuals may be due to low antibody levels, low cellular responses, mismatches between cellular and humoral responses to the parent strain and the variants, or high exposure to infectious cases. We have quantified the magnitude of antibody and cellular responses to the parent strain and Alpha variant in 4 separate groups: partially vaccinated individuals infected with SARS-CoV-2, fully vaccinated individuals infected with SARS-CoV-2, and uninfected HCs sampled early and late after vaccination.

This study demonstrates several important findings about infections in this vaccinated cohort. The antibody responses to the S and S-RBD antigens were comparable at similar time points among fully vaccinated HCs and in those who developed breakthrough infections. Regardless of whether fully or partially vaccinated, however, SARS-CoV-2 infection was associated with lower levels of neutralizing antibodies to the parent strain. These data are consistent with a recent study reporting an association between lower titers of neutralizing antibodies and breakthrough infections (9) and 2 studies identifying nAb levels as potential correlates of protection (13, 14). Our study complements and extends those findings by showing the presence of robust S peptide-specific T cell responses in infected vaccinated individuals, suggesting that lower neutralization titers are specifically associated with infections in vaccinated individuals, regardless of T cell responses. Our study also further reinforces the impact of the second vaccine dose in boosting nAb responses.

In addition to contributing to a better understanding of infections in vaccinated individuals, this study provides insight into vaccine-induced immune response by evaluating early and late HC groups. First, measures of both cellular and humoral immunity decreased with time following the second dose. Measures of the cellular response, however, did not correlate well with antibody responses. Second, these data demonstrate that IgG titers against the Alpha variant S and S-RBD were higher than those against the parent strain in the early HC group. Interestingly, IgG levels to the Alpha variant spike were markedly lower than those to the parent strain in the late HC group. These findings suggest that while mRNA vaccines initially stimulate robust humoral responses to variant viruses, these responses may be short-lived (15–17). Finally, microneutralization assay results indicated that the nAb response against the parent virus was maintained through 6 months in the late HC group, although the binding IgG response decreased, an observation also seen in a study of vaccinated health care workers followed for 6 months (16). While the nAb responses to the parent virus and the Alpha variant were comparable in the early FV-HC group, the level of neutralizing antibodies against the Alpha variant was significantly lower in the late FV-HC group. This decrease in the nAb response to the Alpha variant over time suggests a waning of vaccine-induced immunity to variants of concern.

The magnitude of the antiviral antibody response and the duration of detectable neutralizing antibodies was assessed, with neutralizing antibodies being detectable longer in convalescent (108 days) than vaccinated (65 days) individuals (14, 18). The nAb titers necessary for protection against infection were much higher than needed for protection from severe disease (13, 14). One important factor that has not been carefully addressed to date is the level of mucosal antibodies present after vaccination, as these antibodies are critical for protection from infection in the upper respiratory tract (19). The presence of anti–SARS-CoV-2 IgG in the nasal tract of infected individuals is inversely correlated with the presence of infectious virus (20, 21) emphasizing the importance of mucosal antibody response for rapid neutralization of SARS-CoV-2.

Samples from infected individuals were collected in the first 8 days after symptom onset. The decrease in nAb titers against the Alpha variant relative to the parent virus observed in the FV-HC group was not observed in the infected groups. Among patients in the FV-I group, infection represented the third exposure to SARS-CoV-2, and a rapid memory response likely contributed to the elevated measures of humoral and cellular immunity reported here. Most infections in the FV-I group were caused by the Alpha variant, which may have resulted in a stimulation of Alpha variant-specific antibody responses that reduced the difference between FV-I and FV-HC groups even further. Consistent with this hypothesis, emerging data on the Omicron variant suggests that multiple exposures (i.e., either 3 doses of mRNA vaccines or a combination of infection and 2 vaccine doses) are needed to develop broad immunity to SARS-CoV-2 variants of concern (22). Since the Alpha variant is antigenically distinct from the parent virus, a boosting effect against the parent virus may not be as strong as that against the Alpha variant, thus, a difference in parent virus nAb levels remained.

There are several limitations to this study. The study had a small sample size, and consistent with having received the vaccination, none of the infected participants were hospitalized, suggesting mild disease. Also, the timing of sample collection in this study allowed for evaluation of breakthrough infections with the Alpha variant, but not the Delta or Omicron variants, which may have different pathologies. In addition, all HCs in the study had received 2 doses of vaccine, while infected cases were either fully or partially vaccinated, meaning that the control group for the PV-I is imperfect. While the timeframe of this nonlongitudinal, convenience sample collection relative to vaccination overlapped for the FV-I and late FV-HC group, the late FV-HC group was sampled slightly longer after vaccination, on average, than the FV-I group. Because of this, responses may have waned to a greater degree in the FV-HC than the FV-I, thus attenuating differences between the 2 groups. Finally, low availability of PBMC from HC groups did not allow testing of T cell responses against the Alpha variant, such that data comparing T cell responses to the parent and Alpha strains in infected participants must be interpreted with caution.

Overall, the study demonstrated that humoral and cellular responses decreased with time from vaccination date, potentially increasing the likelihood of infections. It is critically important to understand the magnitude and duration of the protective immune response induced by vaccination and boosting to determine how best to end the COVID-19 pandemic.

## Methods

### Study participants, blood sample processing, and storage

From an outpatient trial recruiting symptomatic newly infected patients, which did not exclude vaccinated individuals, we identified 13 fully vaccinated (more than 14 days after the second vaccination) patients who developed symptomatic breakthrough SARS-CoV-2 infection (FV-I) and 22 partially vaccinated (more than 14 days after the first vaccination) patients who developed symptomatic SARS-CoV-2 infection (PV-I). We then compared these patients to 22 fully vaccinated noninfected health care workers (i.e., HCs evaluated 1–2 weeks after vaccination (Early FV-HC) and 15 fully vaccinated uninfected health care workers evaluated at 5 months after vaccination (Late FV-HC). All study participants had received either BNT162b2 (Pfizer) or mRNA-1273 (Moderna) mRNA vaccines. For those with SARS-CoV-2 infection, clinical symptom information, nasal swabs, and serum samples were obtained as close to onset of symptoms as possible. Demographic and clinical data was self-reported by the research participants.

### SARS-CoV-2 genome sequencing

Automated nucleic acid extraction was performed as described previously (20, 21) using the chemagic 360 (PerkinElmer) following the manufacturer’s protocol. Whole genome sequencing and analysis were performed as previously described (23).

For a subset of samples, 25 ng of RNA, previously extracted using QIAGEN’s Viral RNA mini kit, was processed following the Illumina RNA Prep with Enrichment (L) Tagmentation protocol with Illumina Respiratory Virus Oligo Panel for single-plex enrichment. Libraries were sequenced on the Illumina MiSeq (2 × 76 bp) or iSeq (2 × 151 bp) platform. FASTQ files were analyzed in Illumina’s BaseSpace using the DRAGEN Pathogen Detection application to generate consensus files. The pangolin web-based application, Phylogenetic Assignment of named Global Outbreak LINeages (PANGOLIN) (https://pangolin.cog-uk.io/) was used to identify the SARS-CoV2 lineages from these consensus sequences. Nextclade (https://clades.nextstrain.org/) was used for clade assignment, sequence quality check, and phylogenetic tree construction.

### Expression and purification of parent strain and Alpha variant S- and S-RBD

#### Plasmid preparation.

The plasmids expressing recombinant S and S-RBD for the vaccine strain of SARS-CoV-2 have been described previously (24). The sequence from the SARS-CoV-2 Alpha variant hCoV19/USA/MD-HP01101/2021 (EPI_ISL_825013) was used to engineer S and S-RBD expression plasmids. The Alpha variant S gene was synthesized in its entirety (GeneScript) before cloning into the pCAGGS vector. Site directed mutagenesis was used to introduce a N501Y substitution into the plasmid expressing the S-RBD from the vaccine strain. The plasmids were extracted using GigaPrep kits (Thermo Fisher Scientific) and eluted in molecular biology grade water.

#### Protein purification.

Protein purification by immobilized metal affinity chromatography (IMAC) and gravity flow was adapted from previous methods (24). After washing with PBS (Thermo Fisher Scientific), nickel nitrilotriacetic acid (Ni-NTA) agarose (QIAGEN) was added to the culture supernatant, followed by overnight incubation for 12–16 hours at 4°C on a rotator. For every 150 mL of culture supernatant, 2.5 mL of Ni-NTA agarose was added. Then, 5 mL gravity-flow polypropylene columns (QIAGEN) were equilibrated with PBS. One polypropylene column was used for every 150 mL of culture supernatant. The supernatant-agarose mixture was then loaded onto the column to retain the agarose beads, with recombinant proteins bound to the beads. Each column was then washed, first with 1 × culture supernatant volume of PBS and then with 25 mL of 20 mM imidazole (MilliporeSigma) in PBS wash buffer to remove host cell proteins. Recombinant proteins were then eluted from each column in 3 fractions with 5 mL of 250 mM imidazole in PBS elution buffer per fraction, giving a total of 15 mL eluate per column. The eluate was subsequently dialyzed several times against PBS using Amicon Ultra Centrifugal Filters (MilliporeSigma) at 5000 *g* for 20 minutes at 10°C to remove the imidazole and concentrate the eluate. Filters with a 10 kDa molecular weight cutoff were used for the RBD eluate, whereas filters with a 50 kDa molecular weight cutoff were used for the full-length S protein eluate. The final concentration of the recombinant S and S-RBD proteins was measured by bicinchoninic acid (BCA) assay (Thermo Fisher Scientific) and purity was assessed on 10% SDS-PAGE gels (Bio-Rad) followed by Coomassie blue staining. After sufficient destaining in water overnight, clear single bands were visible for S- and S-RBD proteins at their respective molecular sizes.

For the scale-up purification, preparative IMAC chromatography was performed using an Äkta Explorer 100 (Amersham Biosciences) controlled by Unicorn 5.31 software. HisTrap excel (1 mL) prepacked columns (Cytiva) were used for generating the purified S and S-RBD proteins. For the S-RBD purification process, the equilibration step was performed with PBS buffer for 5 column volumes (CV) at 1 mL/min, followed by loading of 40 CV of the harvest material at 1 mL/min. A wash step with 20 mM imidazole in PBS was performed for 20 CV at 1 mL/min, immediately followed by the step gradient elution of the bound proteins using 15 CV of 500 mM imidazole in PBS at 1 mL/min. During this step, 1 mL fractions were collected and stored for further purity analysis. The column was then reequilibrated with PBS, regenerated using 0.5 M NaOH for 10 CV at 1 mL/min, and finally stored at 20% v/v ethanol. For the S protein purification process, a similar setup was used with slight modifications in the purification protocol. The flow rate during the loading step was reduced to 0.5 mL/min to increase the residence time during loading, thereby increasing the yield of the target protein. The buffer compositions were optimized to enhance the purity in the elution step. This included an addition of 0.4 M NaCl in the equilibration, wash, and elution buffers to help mitigate the electrostatic interactions of contaminants with the tagged protein or resin. The imidazole concentration in the wash buffer was also increased to 30 mM to help remove the tagged contaminants from the elution fractions. SDS-PAGE analysis (Any kDa gel, Bio-Rad) was performed followed by silver staining to analyze the purity of these fractions. The pure fractions were then pooled and buffer-exchanged against PBS (as described above) to generate approximately 10 × concentrated S and RBD protein solutions.

### Viruses and cells

Vero-E6-TMPRSS2 cells (25) were cultured in complete media (CM) consisting of DMEM containing 10% FBS (Gibco, Thermo Fisher Scientific), 1 mM glutamine (Invitrogen, Thermo Fisher Scientific), 1 mM sodium pyruvate (Invitrogen, Thermo Fisher Scientific), 100 U/mL penicillin (Invitrogen, Thermo Fisher Scientific), and 100 μg/mL streptomycin (Invitrogen, Thermo Fisher Scientific). Cells were incubated in a 5% CO2 humidified incubator at 37°C.

The SARS-CoV-2/USA-WA1/2020 virus was obtained from BEI Resources. The Alpha variant of SARS-CoV-2 (hCoV19/USA/MD-HP01101/2021, EPI_ISL_825013) was isolated on Vero-TMPRSS2 cells plated in 24-well dishes and grown to 75% confluence. The CM was removed and replaced with 150 μL of infection medium (IM), which is identical to CM but with the FBS reduced to 2.5%, and 150 μL of the viral transport media containing a swab from a patient confirmed to be SARS-CoV-2 positive added to the culture. The cultures were incubated at 37°C for 2 hours, the inoculum was aspirated and replaced with 0.5 mL of IM, and the cells cultured at 37°C for up to 5 days. When a cytopathic effect was visible in most of the cells, the IM was harvested and stored at –70°C. The presence of SARS-CoV-2 was verified by extracting RNA from the harvested supernatant using the QIAGEN Viral RNA extraction kit and viral RNA was detected using quantitative RT-PCR (26). The consensus sequence of the virus isolate did not differ from the sequence derived from the clinical specimen. The infectious virus titer was determined on Vero-TMPRSS2 cells using a 50% tissue culture infectious dose (TCID50) assay as previously described for SARS-CoV-2 (27, 28). Serial 10-fold dilutions of the virus stock were made in IM, and 100 μL of each dilution was then added to the cells in a 96-well plate in sextuplicate. The cells were incubated at 37°C for 4 days, visualized by staining with naphthol blue-black, and scored visually for cytopathic effect. A Reed and Muench calculation was used to determine the TCID50 per mL (29).

### ELISA

The ELISA protocol was adapted from a protocol published by the Florian Krammer laboratory (24). The 96-well plates (Immulon 4HBX, Thermo Fisher Scientific) were coated with either full-length S protein or S-RBD of the parent strain or the Alpha variant at a volume of 50 μL of 2 μg/mL diluted antigen in filtered, sterile 1 × PBS (Thermo Fisher Scientific) at 4°C overnight. Coating buffer was removed, and the plates were washed 3 times with 300 μL 1 × PBS plus 0.1% Tween-20 (PBST) wash buffer (Thermo Fisher Scientific) and then blocked with 200 μL PBST with 3% nonfat milk (milk powder, American Bio) by volume for 1 hour at room temperature. All plasma samples were heat-inactivated at 56°C on a heating block for 1 hour before use. Negative control samples were prepared at 1:10 dilutions in PBST in 1% nonfat milk and plated at a final dilution of 1:100. A mAb against the SARS–CoV-2 S protein was used as a positive control (1:5000 dilution; Sino Biological, 40150- D001). Plasma samples were prepared in 3-fold serial dilutions starting at 1:20 in PBST in 1% nonfat milk. Blocking solution was removed, and 100 μL diluted plasma was added in duplicate to the plates and incubated at room temperature for 2 hours. Plates were washed 3 times with PBST wash buffer, and 50 μL of secondary antibody was added to the plates and incubated at room temperature for 1 hour. Antihuman secondary antibody, Fc-specific total IgG HRP (1:5000 dilution; Thermo Fisher Scientific, Invitrogen, A18823), was prepared in PBST plus 1% nonfat milk. Plates were washed, and all residual liquid was removed before addition of 100 μL SIGMAFAST OPD (o-phenylenediamine dihydrochloride) solution (MilliporeSigma) to each well, followed by incubation in darkness at room temperature for 10 minutes. To stop the reaction, 50 μL 3M HCl (Thermo Fisher Scientific) was added to each well. The OD of each plate was read at 490 nm (OD490) on a SpectraMax i3 ELISA Plate Reader (BioTek Instruments). A cutoff value for each plate was calculated by summing the average of the OD values of the negative controls and 3 times the standard deviations of the OD values of the negative controls. This cutoff value was subtracted from all sample OD values and negative values set to zero. Background-subtracted OD values were then plotted against the dilution factor to calculate the AUC. For all ELISA data, a titer of 1:180 was determined as a cutoff for positivity using prepandemic and convalescent samples. On the AUC scale, this cutoff was established by taking the average AUC values of samples with a titer of 1:180.

### Microneutralization assay

Plasma nAbs were determined as described for SARS-CoV-2 (30). Two-fold dilutions of plasma (starting at a 1:20 dilution) were made in IM. Infectious virus was added to the plasma dilutions at a final concentration of 1 × 10^4^ TCID_50_/mL (100 TCID_50_ per 100 μL). The samples were incubated for 1 hour at room temperature, and then 100 μL of each dilution was added to 1 well of a 96-well plate of VeroE6-TMPRSS2 cells in hexaplicate. The cells were incubated for 6 hours at 37°C, 5% CO_2_. The inocula were removed, fresh IM was added, and the cells were incubated at 37°C, 5% CO_2_ for 2 days. The cells were fixed by the addition of 100 μL of 4% formaldehyde per well, incubated for at least 4 hours at room temperature, and then stained with Napthol Blue Black (MilliporeSigma). The nAb titer was calculated as the highest serum dilution that eliminated the cytopathic effect in 50% of the wells (NT50) and the AUC was calculated using GraphPad Prism.

### T cell interferon response to SARS-CoV-2 S peptide

#### Peptides and ELISPOT assays.

Peptides for the S protein of SARS-CoV-2 were obtained from BEI Resources and reconstituted with DMSO at a concentration of 10 mg/mL. The SARS-CoV-2 peptides are 12, 13, or 17 mer, with 10 amino acid overlaps. The S protein peptide pool consisted of 181 peptides. The peptides were combined into 1 pool for each viral protein at 10 μg/mL. The Alpha variant S peptides (15 mers with 11 amino acid overlaps) were purchased from JPT Peptide Technologies and used at a concentration of 1ug/mL. For comparison, parent virus S peptides from the same company were used at the same concentration. Stimulation with 1 μg/mL of anti-CD3 antibody (Mabtech) was used as a positive control for each study participant.

*IFN-γELISPOT* assays were performed as previously described (31). Briefly, ELISPOT Pro and ELISPOT Plus kits with precoated plates were purchased from Mabtech. The wells were plated with unfractionated PBMCs at 250,000 cells/well, and the cells were cultured for 20 hours with the SARS-CoV-2 peptide pool. The plates were then processed according to the manufacturer’s protocol and read by a blinded independent investigator using an automated reading system. Four replicates per pool were run, and the mean of replicates was plotted. The replicate farthest from the median was not used. If 2 values were equally distant from the median, then the higher value was discarded. SFUs per million PBMCs were calculated by multiplying SFUs generated by the automated plate reader by 4 (i.e., SFU/250,000 cells were multiplied to yield the standard SFU/million). A response was counted as positive only if treatment induced at least a 3-fold increase and the SFU exceeded 20 spots/million.

### Statistics

AUC values were log_10_ transformed to achieve a normal distribution. Immune read-outs between groups and viruses were compared by 2-tailed *t* tests, paired *t* tests, or 1-way ANOVA with Tukey’s correction for multiple comparisons where appropriate. A *P* value of less than 0.05 was considered statistically significant. Correlations between IgG antibody, microneutralizing assay, and ELISpot assays were assessed using Pearson’s R. Percent changes were calculated as 100*([initial value – final value]/initial value) using log_10_-transformed values. Analyses were performed using GraphPad Prism 8 (GraphPad Software) and Stata 15 (StataCorp).

### Study approval

The study called Convalescent Plasma to Limit SARS-CoV-2 Associated Complications (CSSC-004) was a phase 2 double-blinded randomized control trial with either high titer SARS-CoV-2 convalescent plasma or placebo control plasma. This study was designed as a separate protocol under Johns Hopkins University Investigational New Drug application (19725) and filed as NCT04373460 at clinicaltrials.gov. Johns Hopkins approved the protocol IRB00247590 and acted as the central IRB for the study.

## Author contributions

KAG, DS, JNB, SLK, AP, OL, AC, ALC, and AART conceived and designed the study; DS, EMB, AC, S Shoham, and AART wrote the IRB protocol; KAG, TJG, BP, PB, GM, SLH, ESS, AS, EMB, DH, and DS recruited participants; DS coordinated the collection of all samples; MT cataloged and organized samples and assisted with blinded data organization; HSP, JRS, BAW, CCG, KER, AEJ, IS, HHM, AD, S Suwanmanee, and CC carried out all experiments; PH, LA, NIM, SK, SMC, and MJB produced and purified recombinant SARS-CoV-2 proteins; JS performed statistical analyses; HSP, JRS, SLK, AART, JNB, AP, DS, and KAG wrote the manuscript with substantial input from all coauthors. For co–first authorship, HSP is named first for antibody level determinations and data analysis, followed by JS for coordinating data analysis and IS for virus nAb determinations and data analysis. Order of co–senior authors was predetermined to alternate on collaborative work.

## Supplementary Material

Supplemental data

## Figures and Tables

**Table 1:**
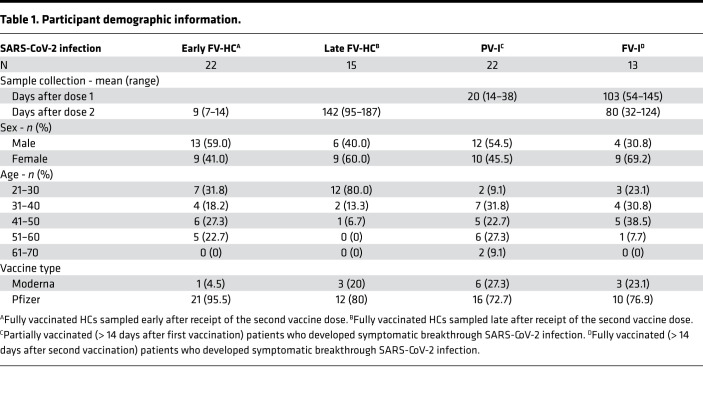
Participant demographic information.

## Version 1 02/01/2022

In-Press Preview

## Version 2 03/08/2022

Electronic publication
