# Supplementary material for: Adaptive immune responses in vaccinated patients with symptomatic SARS-CoV-2 Alpha infection
Source: JCI Insight. 2022 Mar 8;7(5):e155944. doi: 10.1172/jci.insight.155944 (PMC8983140; doi:10.1172/jci.insight.155944)
Supplement: Supplemental data [file jciinsight-7-155944-s210.pdf]

Adaptive immune responses in vaccinated patients with symptomatic SARS-CoV-2 Alpha infection

Supplemental Materials

Supplement Table 1. Samples numbers used in assays.

Group	Early FV-HC	Late FV-HC	PV-I	FV-I
**N**	22	15	22	13
**Data sources - n (%)**				
ELISA	22 (100)	15 (100)	22 (100.0)	13 (100.0)
Neutralizing	22 (100)	15 (100)	22 (100.0)	13 (100.0)
ELISpot (vaccine virus)	15 (68)	13 (87)	11 (50.0)	8 (61.5)
**Complete data**	15 (68)	13 (87)	11 (50)	8 (61.5)

Supplemental Table 2. Geometric mean titers

GMT (95% CI)	Early FV-HC	Late FV-HC	PV-I	FV-I
**Parent S IgG**	35820 (20992 - 61122)	10109 (6509 - 15702)	27906 (15394 - 50586)	20444 (9327 - 44812)
**Parent S-RBD IgG**	43740 (25439 - 75208)	2705 (1721 - 4252)	12551 (6717 - 23453)	14580 (6188 - 34354)
**Alpha S IgG**	35820 (25915 - 49511)	3901 (1807 - 8422)	7618 (4202 - 13809)	6262 (2888 - 13579)
**Alpha S-RBD IgG**	102226 (70886 - 147423)	5229 (2790 - 9802)	10279 (5303 - 19925)	18787 (7303 - 48330)
**Parent nAb**	181 (121 - 273)	202 (132 - 309)	18 (12 - 26)	38 (20 - 71)
**Alpha nAb**	206 (140 - 303)	76 (51 - 114)	21 (13 - 34)	47 (24 - 93)

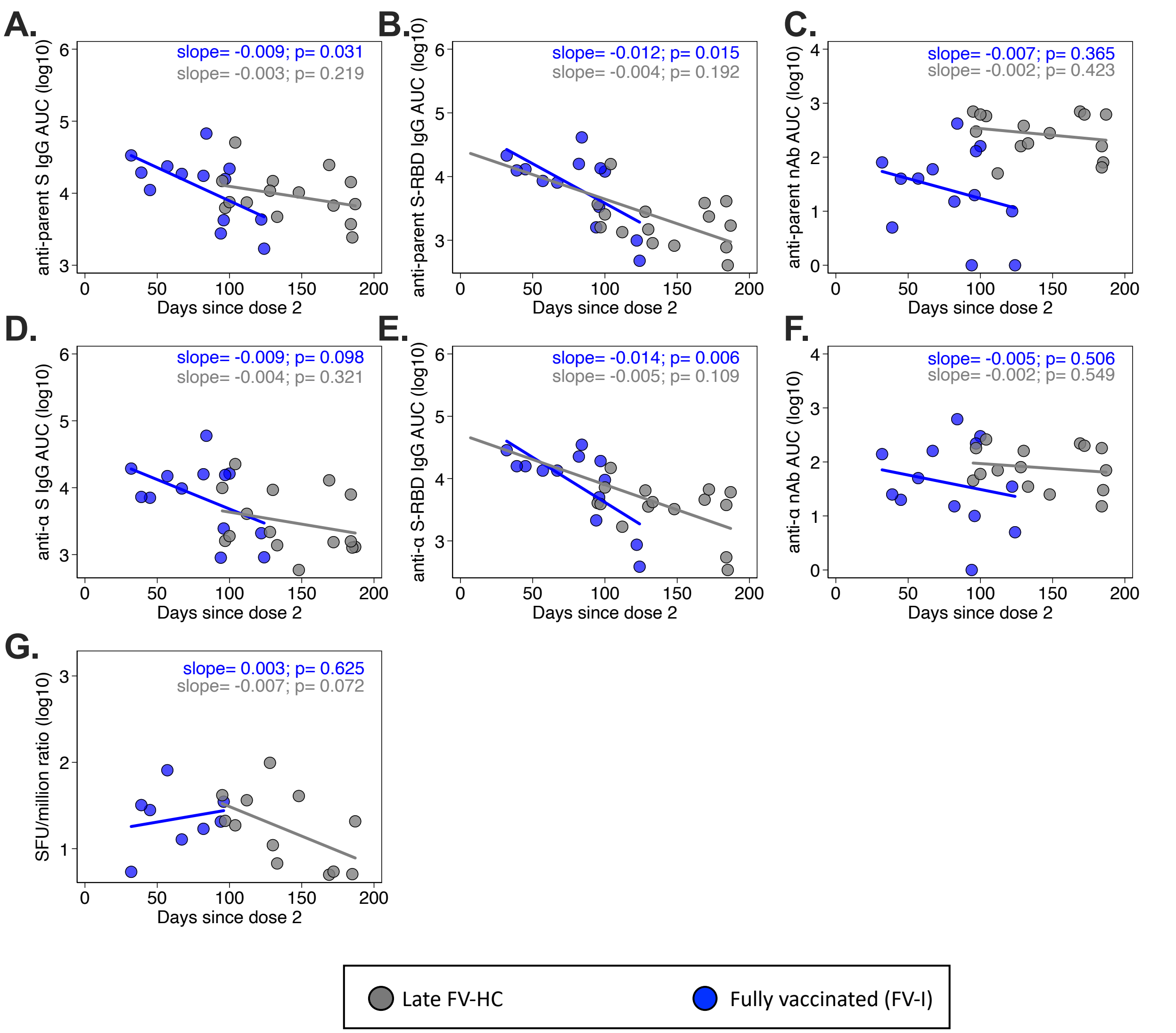

Supplemental figure 1. Measures of humoral and cellular immunity tend to decline with time after vaccination, regardless of infection status. Humoral immune responses are shown as the parent strain anti-S-IgG AUC (**A**), anti-S-RBD-IgG AUC (**B**), and neutralizing antibody (nAb) AUC (**C**) versus days since receipt of the second dose of a SARS-CoV-2 mRNA vaccine for individuals with breakthrough infection (n = 13) and healthy controls without evidence of breakthrough infection (n = 15). Humoral immune responses to the alpha variant are shown as anti-S-IgG AUC (**D**), anti-S-RBD-IgG AUC (**E**), and neutralizing antibody (nAb) AUC (**F**). Similarly, measures of cellular immunity are shown as a ratio of SFU/million (**G**)) in treated and untreated cells versus days since receipt of the second dose of a SARS-CoV-2 mRNA vaccine. Slopes and associated p-values were derived from simple linear regressions for each outcome for both groups.

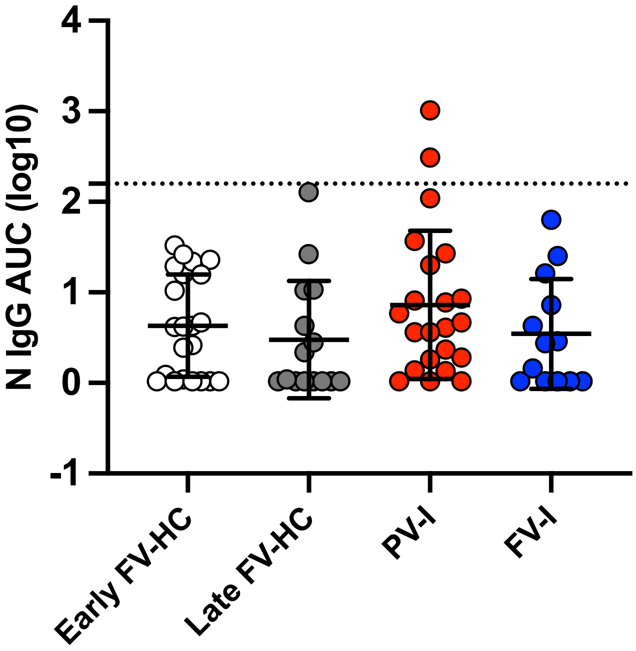

Supplement figure 2. Antibody response to SARS-CoV-2 virus nucleocapsid (N) in vaccinated people. Indirect ELISAs were used to measure IgG against the nucleocapsid protein and results graphed as area under the curve (AUC) values. Data is shown for healthy controls (HCs), with no evidence of SARS-CoV-2 infection 7-14 days (white circles, early FV-HC, n =22) or 95-187 days (gray circles, Late FV-HC, n = 15) after the second dose. Red and blue circles indicate samples obtained from participants who had received either 1 (PV-I, n = 22) or 2 (FV-I, n= 13) vaccine doses, and who had confirmed SARS-CoV-2 infection. The dashed line indicates the limit of detction. A one-way ANOVA with Tukey's correction for multiple comparions was used to compare groups, and, p-values below 0.05 were considered statically significant.
